# Comparison of the measurement properties and consistency between the EQ-5D-3L and EQ-5D-Y-3L in adolescents aged 15–17 in China

**DOI:** 10.1186/s12955-024-02275-6

**Published:** 2024-07-29

**Authors:** Jiefu Wang, Tianqi Hong, Haoran Fang, Chang Luo, Xiaoning He, Shitong Xie

**Affiliations:** 1 Tianjin Medical University Cancer Institute & Hospital, National Clinical Research Center for Cancer, Tianjin’s Clinical Research Center for Cancer, Tianjin Key Laboratory of Digestive Cancer, Tianjin, China; 2 School of Biomedical Engineering, McMaster University, Hamilton, Ontario Canada; 3 School of Pharmaceutical Science and Technology, Faculty of Medicine, Tianjin University, Tianjin, China; 4 Center for Social Science Survey and Data, Tianjin University, Tianjin, China

**Keywords:** Health-related quality of life, EQ-5D-3L, EQ-5D-Y-3L, Consistency, Measurement properties, Adolescent, China

## Abstract

**Objective:**

To evaluate and compare the measurement properties and consistency between the Chinese versions of EQ-5D-3L and EQ-5D-Y-3L among Chinese adolescent populations aged 15–17 years.

**Methods:**

Chinese adolescents aged 15–17 studying in high school were recruited through online survey. Social-demographic characteristics and self-reported EQ-5D-3L and EQ-5D-Y-3L responses were collected in the survey. The consistency of responses between the two measures was assessed using redistribution property, and the consistency of utility values was assessed by intraclass correlation coefficients (ICC). Convergent validity and known-group validity were examined using Spearman’s rank correlation, F-test and effect sizes, respectively. Sensitivity was compared using relative efficiency (RE).

**Results:**

762 respondents (48.8% male; age 15–17 years;) were recruited. The EQ-5D-3L showed a more severe ceiling effect than EQ-5D-Y-3L (78.2% vs. 66.0%). Respondents reported higher proportions of having problems in four dimensions using the EQ-5D-Y-3L than using the EQ-5D-3L. The consistency of corresponding dimensions between the two measures was relatively good, while non-negligible proportions of inconsistency were observed in “pain/discomfort” (11.4%) and “anxiety/depression” (15.7%) dimensions. The ICC of the utility values between the EQ-5D-3L and EQ-5D-Y-3L was 0.852 (*p* < 0.001). The Spearman’s rank correlation (range: 0.385–0.620) indicated an acceptable convergent validity between the correlative dimensions of the EQ-5D-3L and EQ-5D-Y-3L. The EQ-5D-Y-3L had a higher efficiency than the EQ-5D-3L at detecting differences across EQ VAS subgroups (ES = 1.793 for EQ-5D-3L, ES = 1.920 for EQ-5D-Y-3L). Mixed results were observed in sensitivity.

**Conclusions:**

Both the EQ-5D-3L and EQ-5D-Y-3L are demonstrated to be valid and generally consistent for measuring HRQoL among adolescents aged 15–17 years in China. Respondents reported higher proportions of having problems using the EQ-5D-Y-3L than using the EQ-5D-3L. More research is warranted to compare the discriminant validity and test-retest reliability between the two measures.

## Introduction

Health-related quality of life (HRQoL) has been widely used as a multidimensional concept worldwide to assess an individual’s health status based on physical, psychological, and social functioning [1]. HRQoL can be assessed using a generic preference-based measure (GPBM), which consists of a health state description system and corresponding country-specific sets of health utility values derived from a representative sample of the general population [1]. Health utility, which provides a standardized weight to interpret the severity of health states, is measured on a standard scale, with an upper limit of 1 representing full health, 0 representing death, and values below 0 indicating health states that are considered worse than death [2–4]. The EQ-5D, developed by the EuroQol Group, is one of the most widely used GPBMs and is recommended as a standard measure for health technology assessment applications in many countries [5].

In recent years, the development and validation of HRQoL measures specific to children and adolescents have gained popularity [6–8]. The youth version of the EQ-5D, the EQ-5D-Y, is a GPBM designed for measuring the HRQoL of respondents aged 8–15 years. This version retains the same health dimensions as the EQ-5D, with adjustments made to its dimension headings, wording of severity labels, and layout for child/adolescent respondents. Several studies have confirmed that the EQ-5D-Y-3L demonstrate good feasibility, validity, reliability, and sensitivity in measuring HRQoL of children and adolescents aged 8–17 years with or without certain health conditions in several countries and regions, including China [9–17].

According to the instructions in the EQ-5D-Y-3L user guidance published by the EuroQol Group, both the EQ-5D-3L and EQ-5D-Y-3L can be used for adolescents aged 12–15 years, while generally, EQ-5D-Y is recommended. For adolescents aged 16 years and older, the EuroQol Group recommends using an adult version. While, the user guidance also mentioned that “if a study that only includes children up to age 18, in this case, it may be preferable to use EQ-5D-Y across the full age range to avoid using two different versions of EQ-5D” [18]. However, there is currently a lack of comparison of measurement properties and the consistency of responses between the EQ-5D-3L and EQ-5D-Y-3L in adolescents, especially those aged 15–17 years, worldwide. Evidence supporting the selection of a more appropriate measure for adolescents is needed to facilitate a more accurate and sensitive measurement of HRQoL for this population.

Therefore, the aim of this study was to compare the measurement properties and consistency between the EQ-5D-3L and EQ-5D-Y-3L among adolescents aged 15–17 in China.

## Methods

The protocol of this study was approved by the Institutional Review Board of the School of Pharmaceutical Science and Technology, Tianjin University, China (No. 20,230,324). Informed consent was obtained from all included adolescents and their parents or legal guardians.

### Data source

The data used for this analysis were obtained from an online survey (from Aug to Oct 2023) investigating the health status and myopic status of Chinese adolescents aged 15–18 studying in high school. Since it is recommended to use the EQ-5D-Y-3L among children and adolescents, respondents aged ≥ 18 were excluded from the study.

Recruitment of the respondents was conducted through a professional online panel company. The company reached out to eligible panel members and inquired about their willingness to participate through text messages, emails, and push notifications within apps. Adolescents that were willing to participate in were invited to complete the self-reported online survey via computer or mobile phone. The survey collected three parts of information: (1) Information on social-demographic characteristics, including age, sex, ethnicity, and grade in high school; (2) health-related indicators, including sitting posture, weekly total duration of outdoor activities, and myopic status (categorized into mild, moderate, and high myopia following the recommendations of the International Myopia Institute [IMI] [19–21]; and (3) responses of the HRQoL measures including the EQ-5D-3L and EQ-5D-Y-3L, with the random order. A quality control check question by asking respondents the answer to a calculation question “15 + 7 = ?” was also included in the survey. Records giving incorrect answers to the QC question or identified with a duplicate IP address were excluded from this study.

### Instruments

*EQ-5D-3L.* The EQ-5D-3L is one of the most commonly used GPBMs around the world, which consists of the descriptive system and the visual analogue scale (EQ VAS). The descriptive system has five dimensions of HRQoL, namely, mobility (MO), self-care (SC), usual activities (UA), pain/discomfort (PD), and anxiety/depression (AD). The EQ-5D-3L uses three levels of discrimination for each dimension (no problems, some problems, extreme problems). The EQ-5D-3L describes 243 health states that result from combining the three response possibilities on five dimensions. The EQ VAS consists of a visual scale that ranges from the best condition you can imagine to the worst imaginable state, divided into 100 units. EQ VAS is used as a quantitative measure of the perception of one’s health [22]. The Chinese EQ-5D-3L utility value set developed using the time trade-off (TTO) method has a range of utility values from − 0.149 (33,333) to 1 (11,111) [23].

*EQ-5D-Y-3L.* The EQ-5D-Y-3L is a child and adolescent-friendly version of the EQ-5D-3L using the same descriptive system of five dimensions but different wordings (‘mobility [walking about]’, ‘looking after myself’, ‘doing usual activities’, ‘having pain or discomfort’, and ‘feeling worried, sad or unhappy’), with three severity levels (no problems, some problems, extreme problems) in each dimension. The Chinese EQ-5D-Y-3L utility value set developed using the composite time trade-off (cTTO) method has a range of utility values from − 0.089 (33,333) to 1 (11,111) [24]. The EQ-5D-Y-3L also includes a visual analog scale (EQ VAS), which ranges from 0 (the worst imaginable health state) to 100 (the best imaginable health state) [25].

### Statistical analysis

#### Descriptive statistics

To describe the characteristics of the respondents, we used the mean and standard deviation (SD) for continuous variables, as well as the frequency and proportion of categorical variables. We reported the distribution of response levels on each dimension of the EQ-5D-3L and EQ-5D-Y-3L using histograms [26, 27]. Descriptive statistics, including mean and standard deviation (SD), were also calculated for the utility values of the EQ-5D-3L and EQ-5D-Y-3L, as well as the EQ VAS score. The EQ VAS score was used as an indicator of self-reported health status and was divided into four subgroups: <65 (poor), 65–79 (fair), 80–89 (good), and 90–100 (excellent) in this study [28–30].

#### Consistency

Redistribution between the EQ-5D-3L and EQ-5D-Y-3L was evaluated by comparing responses on EQ-5D-3L and EQ-5D-Y-3L corresponding dimensions. We presented the redistribution situation of EQ-5D-3L and EQ-5D-Y-3L using a Sankey diagram, in which the proportions of consistency for each level across the different dimensions were visually reported [15]. Inconsistent response pairs were defined as instances where the EQ-5D-3L response differed from the corresponding EQ-5D-Y-3L response (e.g., an individual endorsed level 1 of the dimension of UA in the EQ-5D-3L while level 2 of the same dimension in the EQ-5D-Y-3L). Besides, the intraclass correlation coefficient (ICC) was used to examine the agreement of health utility values between the EQ-5D-3L and EQ-5D-Y-3L. The ICC was calculated using a two-way mixed effects model based on absolute agreement [31]. An ICC less than 0.5, between 0.5 and 0.75, between 0.75 and 0.90, and greater than 0.90 indicate poor, moderate, good and excellent agreement, respectively [31].

#### Measurement properties

We focused on ceiling and floor effects, convergent validity, known-groups validity, and responsiveness, which are important for evaluating the performance of measurement properties of preference-based measures.

##### Ceiling and floor effects

The ceiling and floor effects for both the EQ-5D-3L and EQ-5D-Y-3L were assessed by calculating the percentage of respondents reporting “no problems” or reporting “extreme problems” across all five dimensions, that is, the percentage of the state of “11111” or “33333”. A ceiling or floor effect was considered present if more than 15% of respondents achieved the maximum or minimum of the scale [32].

##### Convergent validity

The convergent validity was assessed by calculating the Spearman rank correlation coefficient (r) between dimensions of the EQ-5D-3L and EQ-5D-Y-3L. An absolute coefficient value greater than 0.5 indicates a strong correlation, 0.35–0.49 moderate correlation, 0.2–0.34 weak correlation, and less than 0.2 poor correlation [33–36]. We expected to find significantly strong correlations in the same dimensions between the EQ-5D-3L and EQ-5D-Y-3L [15].

##### Known-groups validity

Known-groups validity was investigated by determining whether groups known to differ in health status could be distinguished by the EQ-5D-3L and EQ-5D-Y-3L utility values [35]. We hypothesized that respondents with a higher degree of myopia and those with poorer self-reported health status would have lower utility values. The F-test was used to analyze potential differences in utility values of the EQ-5D-3L and EQ-5D-Y-3L across different subgroups [15]. Effect size (ES) was also used to define the discriminatory ability of the EQ-5D-3L and EQ-5D-Y-3L, calculated as the difference between two subgroup means divided by the pooled standard deviation [37]. For multi-categorical variables, the effect size was calculated between extreme subgroups (e.g., between the EQ VAS < 65 subgroup and the 90 ≤ EQ VAS ≤ 100 subgroup) [26, 38]. ES (Cohen’s d) was determined by dividing the difference in mean scores by the pooled SD, with thresholds of 0.2 (small), 0.5 (moderate), and 0.8 (large), traditionally [33]. A larger effect size indicates better discriminatory ability of the measure [39].

##### Sensitivity

The sensitivity of the EQ-5D-3L and EQ-5D-Y-3L to detect differences in self-reported health indicators was assessed using the relative efficiency (RE) calculated as the ratio of the t-statistic generated by an analysis of variance test for a single factor [40–42]. The scale with a higher t-statistic is considered more efficient as it is more likely to achieve statistical significance. An RE value of 1.0 indicates that EQ-5D-Y-3L is as efficient as EQ-5D-3L in detecting these external health indicator differences. A value greater than 1 indicates that EQ-5D-Y-3L is more sensitive than EQ-5D-3L, and the opposite is true for values less than 1 [43].

All the statistical analyses were executed using STATA version 16.0, a product of StataCorp LLC, headquartered in College Station, Texas, USA. All the statistical tests reported were two-tailed and conducted at a significance level of 0.05.

## Results

### Descriptive statistics

Among 1496 survey respondents, 423 (28.3%) of them were excluded since they were aged ≥ 18 years, and 311 (20.8%) were excluded due to having missing data. A total of 762 adolescents were included in this study. As shown in Table 1, 48.8% (*n* = 372) of the respondents were male, with a mean (SD) age of 16.4 (0.5) years, ranging from 15 to 17 years. 93.96% (*n* = 716) of adolescents had myopia with different severity level, only 6.04% (*n* = 46) of adolescents had non-myopia. The mean (SD) duration of outdoor activities was 3.8 (3.1) hours per week. The mean (SD) utility value was 0.958 (0.103) for the EQ-5D-3L and 0.959 (0.075) for the EQ-5D-Y-3L. The mean (SD) EQ VAS score was 86.2 (17.6) (EQ VAS scores reported in both the EQ-5D-3L and EQ-5D-Y-3L were consistent among all respondents).

**Table 1:** Characteristics of respondents (*N* = 762)

Characteristic	*N* (%)
**Sex**	
Male	372 (48.82%)
Female	390 (51.18%)
**Ethnic**	
Han	704 (92.39%)
Other	58 (7.61%)
**Age (mean [SD])**	16.416 (0.504)
**Age**	
15 years	4 (0.52%)
16 years	437 (57.35%)
17 years	321 (42.13%)
**Grade level in high school**	
Grade one	543 (71.26%)
Grade two	216 (28.35%)
Grade three	3 (0.39%)
**Myopic status**	
non-myopia	46 (6.04%)
low myopia	211 (27.69%)
moderate myopia	350 (45.93%)
high myopia	155 (20.34%)
**Sitting posture**	
Neutral sitting posture	408 (53.54%)
Prone sitting position	34 (4.46%)
Lateral leaning to the left	119 (15.62%)
Lateral leaning to the right	34 (4.46%)
Indeterminate posture	167 (21.92%)
**Duration of outdoor activities per week (hours**,** mean [SD])**	3.8 (3.1)
**EQ-5D-3L utility value (mean [SD])**	0.959 (0.103)
**EQ-5D-Y-3L utility value (mean [SD])**	0.958 (0.075)
**EQ VAS score (mean [SD])** ^**a**^	86.156 (17.641)
**EQ VAS score**	
90–100	463 (60.76%)
80–89	152 (19.95%)
65–79	80 (10.50%)
< 65	67 (8.79%)

Abbr: SD: Standard deviation

^a^ EQ VAS scores reported in both the EQ-5D-3L and EQ-5D-Y-3L were consistent among all respondents

**Fig. 1:**
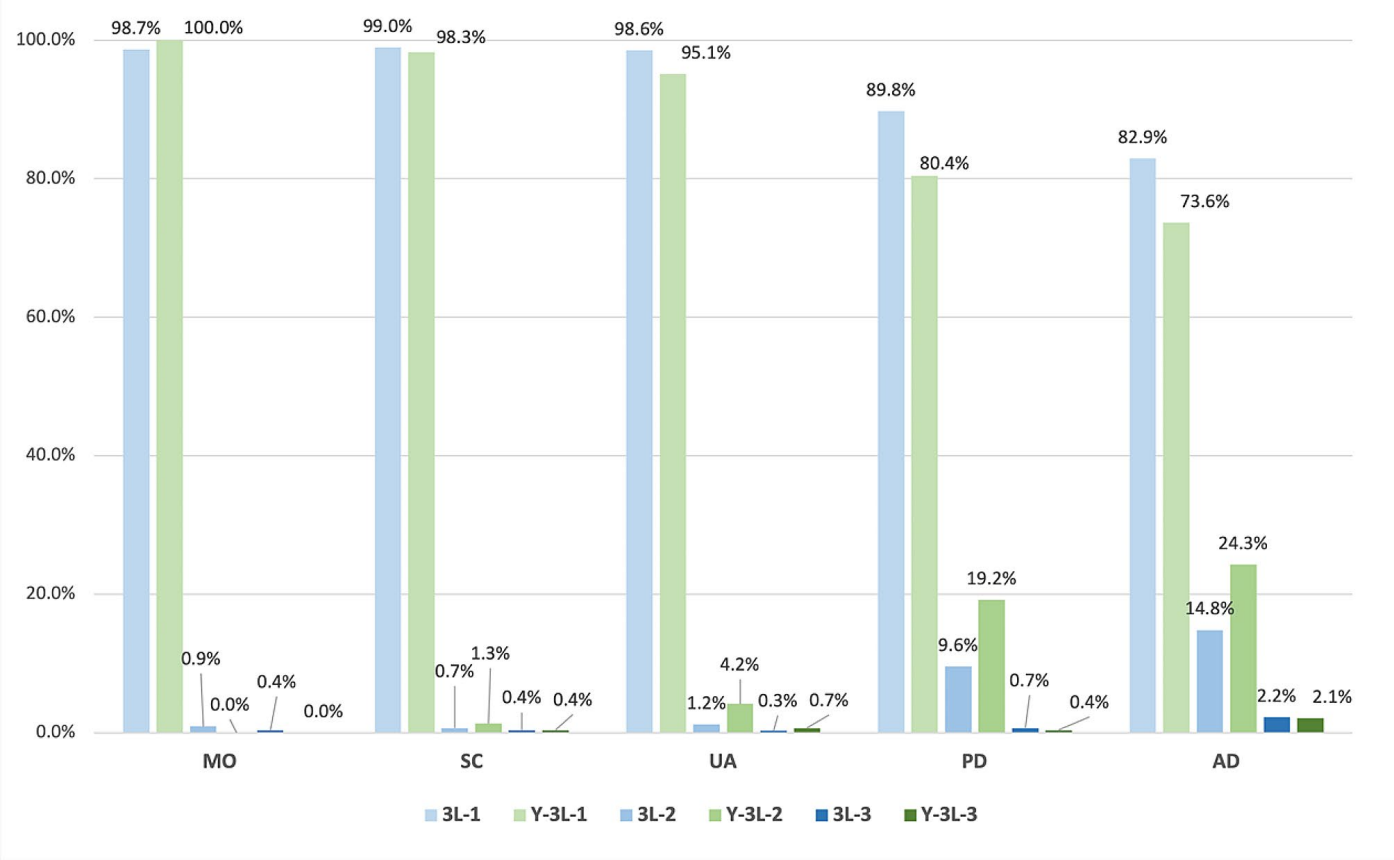
Distribution across dimension-levels of the EQ-5D-3L and the EQ-5D-Y-3L

The distribution of the responses to the EQ-5D-3L and EQ-5D-Y-3L are presented in Fig. 1. For the EQ-5D-3L, the proportion of respondents indicated no problems (level 1) was the highest SC (99.0%), followed by MO (98.7%) UA (98.6%), PD (89.8%), and AD (82.9%). For the EQ-5D-Y-3L, the dimension with the highest proportion of no problems was MO (100.0%), followed by SC (98.3%), UA (95.1%), PD (80.4%), and AD (73.6%). Respondents reported higher proportions of having problems with SC, UA, PD, and AD dimensions using the EQ-5D-Y-3L than using the EQ-5D-3L.

### Consistency

**Fig. 2:**
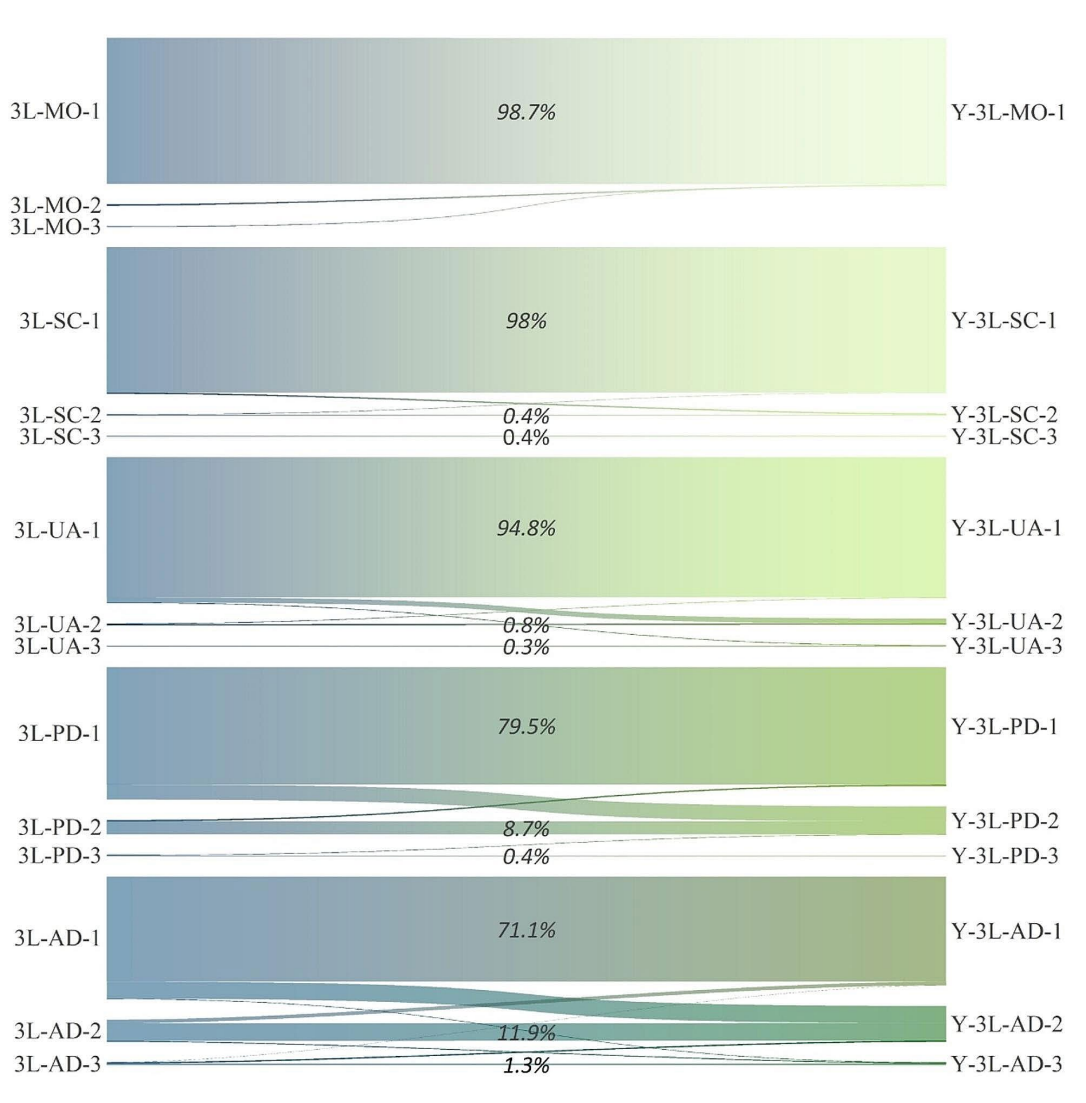
The redistribution properties from EQ-5D-3L to EQ-5D-Y-3L Note: For example, “3L-MO-1” represents “response at level 1 in the mobility dimension of EQ-5D-3L”

The redistribution between the EQ-5D-3L and EQ-5D-Y-3L is presented in Fig. 2. The SC dimension exhibits the highest level of consistency (98.8%). A full level 1 response occurred for the MO dimension in the EQ-5D-Y-3L with an excellent consistency (98.7%). UA dimensions also demonstrated a good consistency of 95.8%. While two levels show moderate levels of consistency in the pain/discomfort (88.6%). Approximately 10.2% of respondents indicated a level 1 on the EQ-5D-3L but reported a level 2 on the EQ-5D-Y-3L. Conversely, 0.9% of individuals reported a level 2 on the EQ-5D-3L yet indicated a level 1 on the EQ-5D-Y-3L. Additionally, a minority of 0.3% of participants rated a level 3 on the EQ-5D-3L while reporting a level 2 on the EQ-5D-Y-3L. AD dimension showed the lowest consistency (84.4%) in this study. 11.5% and 0.3% of respondents indicated a level 1 on the EQ-5D-3L but reported a level 2 and level 3 on the EQ-5D-Y-3L; 2.4% and 0.5% of individuals reported a level 2 on the EQ-5D-3L, yet indicated a level 1 and level 3 on the EQ-5D-Y-3L. Additionally, a minority of 0.1% and 0.8% of participants rated a level 3 on the EQ-5D-3L, while reporting a level 1 and level 2 on the EQ-5D-Y-3L.

The ICC between the utility values of the EQ-5D-3L and EQ-5D-Y-3L was 0.852 (*p* < 0.001), indicating a good agreement.

### Measurement properties of the EQ-5D-3L and EQ-5D-Y-3L

#### Ceiling and floor effects

Both the EQ-5D-3L and EQ-5D-Y-3L exhibited significant ceiling effects, as shown in the response distributions depicted in Fig. 1. The proportion of respondents reporting the best health state was 78.2% (*n* = 596) for EQ-5D-3L and 66.0% (*n* = 503) for the EQ-5D-Y-3L. No significant floor effects were observed for either measure.

#### Convergent validity

As shown in Table 2, most dimensions of the EQ-5D-3L and EQ-5D-Y-3L showed a positive correlation, with Spearman rank correlation coefficients ranging from 0.124 to 0.620. As expected, the correlations between MO, SC, PD, and AD dimensions of the EQ-5D-3L and its corresponding dimension in EQ-5D-Y-3L were the highest among all correlations between that dimension of EQ-5D-3L and EQ-5D-Y-3L. The dimensions SC, PD, and AD were highly correlated with their counterparts in the other instrument (*r* = 0.587, *r* = 0.613, *r* = 0.620, respectively; *p* < 0.001). The dimension of UA showed a moderate correlation with its counterpart in the other instrument (*r* = 0.385, *p* < 0.001). Furthermore, the SC dimension of the EQ-5D-Y-3L exhibits the strongest correlation with the UA dimension of the EQ-5D-3L, which contradicts our initial prediction. Due to the single-level option for the MO dimension in the EQ-5D-Y-3L, we were unable to determine the correlation between each dimension of EQ-5D-3L and MO dimension in the EQ-5D-Y-3L.

**Table 2:** Correlations between EQ-5D-3L and EQ-5D-Y-3L dimensions

EQ-5D-Y-3L	EQ-5D-3L				
	Mobility	Self-care	Usual activities	Pain/ Discomfort	Anxiety/ Depressed
Mobility (walking about)	/	/	/	/	/
Looking after myself	0.433^***^	0.587^***^	0.413^***^	0.236^***^	0.161^***^
Doing usual activities	0.355^***^	0.343^***^	0.385^***^	0.353^***^	0.249^***^
Having pain or discomfort	0.186^***^	0.124^***^	0.228^***^	0.613^***^	0.363^***^
Feeling worried, sad or unhappy	0.182^***^	0.125^***^	0.198^***^	0.374^***^	0.620^***^

Note: * *p* < 0.05; **p < 0.01; *** *p* < 0.001

“/“ in the Mobility (walking about) dimension attributed to the single-level option; Spearman’s correlation was used

#### Known-group validity

As shown in Table 3, there were no significant differences in utility values between the EQ-5D-3L and EQ-5D-Y-3L across groups defined by demographic characteristics, socioeconomic status, and myopia-related indicators. Small effect sizes (< 0.2) were not reached in most subgroups, including sex, ethnic, and myopic group. However, in the EQ VAS subgroup, both the EQ-5D-3L and EQ-5D-Y-3L showed large effect sizes (> 0.8). The F-statistic revealed significant differences in utility values between EQ VAS groups for both the EQ-5D-3L and EQ-5D-Y-3L (*p* < 0.001), and the effect size for the EQ-5D-Y-3L (1.920) was larger than that for the EQ-5D-3L (1.793).

**Table 3:** Discriminative capacity and univariate analyses for EQ-5D-3L and EQ-5D-Y-3L utility values within different groups (*N* = 762)

	Groups	*N*	Utility value (Mean [SD])	F-statistic	*p* value	Effect size (95% CI)
EQ-5D-3L	**Sex**					
	Male	372	0.927 (0.113)	0.04	0.833	-0.015 (-0.157,0.127)
	Female	390	0.929 (0.065)			
EQ-5D-Y-3L	**Sex**					
	Male	372	0.953 (0.080)	1.86	0.173	0.099 (-0.043,0.241)
	Female	390	0.946 (0.061)			
EQ-5D-3L	**Ethnic**					
	Han	704	0.928 (0.093)	0.17	0.683	-0.056 (-0.323, 0.212)
	Another	58	0.933 (0.080)			
EQ-5D-Y-3L	**Ethnic**					
	Han	704	0.950 (0.071)	0.02	0.889	-0.019 (-0.287,0.248)
	Another	58	0.951 (0.066)			
EQ-5D-3L	**Education**					
	Grade one	543	0.932 (0.082)	2.20	0.112	-0.352 (-1.485,0.782)
	Grade two	216	0.917 (0.112)			
	Grade three	3	0.961 (0.000)			
EQ-5D-Y-3L	**Education**					
	Grade one	543	0.953 (0.065)	2.28	0.103	0.015 (-1.118,1.148)
	Grade two	216	0.941 (0.084)			
	Grade three	3	0.952 (0.032)			
EQ-5D-3L	**Myopic group**					
	Non-myopia	46	0.907 (0.173)	1.9	0.128	-0.196 (-0.524,0.133)
	Low myopia	211	0.921 (0.112)			
	Moderate myopia	350	0.935 (0.064)			
	High myopia	155	0.928 (0.078)			
EQ-5D-Y-3L	**Myopic group**					
	Non-myopia	46	0.944 (0.115)	0.8	0.492	-0.061 (-0.389,0.267)
	Low myopia	211	0.945 (0.081)			
	Moderate myopia	350	0.954 (0.059)			
	High myopia	155	0.949 (0.062)			
EQ-5D-3L	**EQ VAS**					
	90–100	463	0.954 (0.026)	69.93	**< 0.001**	1.647 (1.372,1.921)
	80–89	152	0.926 (0.063)			
	65–79	80	0.873 (0.099)			
	< 65	67	0.817 (0.224)			
EQ-5D-Y-3L	**EQ VAS**					
	90–100	463	0.975 (0.035)	87.72	**< 0.001**	1.900 (1.619,2.179)
	80–89	152	0.934 (0.062)			
	65–79	80	0.911 (0.064)			
	< 65	67	0.860 (0.142)			

One-way analyses were performed to identify statistically significant effects of variables on utility values

The effect size was calculated as the difference between the mean utility of two sub-groups divided by the pooled standard deviation

95% CI: 95% confidence interval; SD: standard deviation

Low myopia SE < − 0.5 to > − 3.00 diopter [D]; Moderate myopia SE ≤–3.00 to > − 6.00 D; High myopia SE ≤ − 6.00 D

#### Sensitivity

As shown in Table 4, In terms of differences between self-reported health status groups categorized as “excellent” or “poor,” the efficiency of the EQ-5D-Y-3L was higher with REs ranged from 2.2 to 18.8% (Table 4). However, when groups were divided into two categories based on “good” self-reported health status, it was found that the EQ-5D-3L had a higher efficiency in detecting differences in self-reported health status with a RE of 5.7%.

**Table 4:** Sensitivity of EQ-5D-3L and EQ-5D-Y-3L to detect differences in different self-reported health status groups (*N* = 762)

	Categorization of different self-reported health status groups	*N*	Utility value (Mean [SD])	Effect size	t-statistic	Relative efficiency (RE)
				(95% CI)		
EQ-5D-3L	Excellent	463	0.991 (0.036)	0.862 (0.710, 1.013)	11.624	1.000
	Good, Fair, Bad	299	0.909 (0.146)			
EQ-5D-3L	Excellent, Good	615	0.981 (0.053)	1.288 (1.096, 1.479)	14.044	1.000
	Fair, Bad	147	0.863 (0.181)			
EQ-5D-3L	Excellent, Good, Fair	695	0.971 (0.057)	1.464 (1.202, 1.724)	11.455	1.000
	Bad	67	0.831 (0.234)			
EQ-5D-Y-3L	Excellent	463	0.985 (0.039)	1.024 (0.869, 1.177)	13.809	1.188
	Good, Fair, Bad	299	0.916 (0.095)			
EQ-5D-Y-3L	Excellent, Good	615	0.974 (0.051)	1.241 (1.024, 1.404)	13.241	0.943
	Fair, Bad	147	0.892 (0.113)			
EQ-5D-Y-3L	Excellent, Good, Fair	695	0.967 (0.057)	1.496 (1.234, 1.757)	11.705	1.022
	Bad	67	0.864 (0.146)			

Effect size: ES represents Hedges’s g, which is calculated by dividing the difference in means between two sets of data by their SSD

RE of EQ-5D-3L is presented, and reference is EQ-5D-Y-3L, of which RE is 1.000

T-tests were performed to identify statistically significant effects of dichotomous variables on utility values

95% CI: 95% confidence interval; SD: standard deviation

## Discussion

Both the EQ-5D-3L and EQ-5D-Y-3L have been widely applied in populations with specific conditions [9, 10, 44–49], yet there is a paucity of evidence comparing their measurement properties and consistency of responses in adolescent populations, especially in adolescents aged 15–17 years. To the best of our knowledge, this is the first study to examine the measurement properties and consistency of responses of the EQ-5D-3L and EQ-5D-Y-3L among adolescents aged 15–17 in China. This study could aid healthcare or public health professionals and regulators in understanding and selecting appropriate measures for clinical interventions and policy decisions concerning adolescent conditions.

Both the EQ-5D-3L and EQ-5D-Y-3L detected considerable ceiling effects (78.2% vs. 66.0%). The significant ceiling effect of the EQ-5D-3L, ranging from 67.0 to 42.1%, has been demonstrated in previous studies [50–55]. For the EQ-5D-Y-3L, the ceiling effects ranged from 37.0 to 68.8% in previous studies, which also shows a significant ceiling effect [12, 56–59]. Compared with the EQ-5D-3L, a lower ceiling effect for the EQ-5D-Y-3L could be explained by the updated wording of EQ-5D-Y-3L being more suitable for adolescents, providing a clearer understanding of the questionnaire, which elicited a more relevant response [60].

Although the responses of the same dimensions between the EQ-5D-3L and EQ-5D-Y-3L were relatively comparable, we did observe some inconsistencies. We found that the inconsistency proportions of PD and AD dimensions were 11.4% and 15.6%. This indicated that more adolescents reported problems with these two dimensions when responding to the EQ-5D-Y-3L, which is consistent with the previous research findings of Jennifer Jelsma [61]. A possible reason may be that the two instruments present different examples within the stems of the corresponding dimensions; for instance, the AD dimension in the EQ-5D-3L describes “anxiety (e.g., nervousness, worry, restlessness, etc.) / depression (e.g., lack of interest in doing things, feeling down, etc.)”, while the corresponding dimension in the EQ-5D-Y-3L merely states “feeling worried, sad or unhappy” [18, 22, 60]. The difference in descriptions between the two instruments could lead to interpretation biases among adolescents, who may find it difficult to distinguish between levels 1 and 2 in these two dimensions.

High correlations were found in most of the dimensions between the two instruments, except the UA dimension. A moderate correlation (*r* = 0.377, *p* < 0.001) was observed in the counterpart dimension between the two instruments. A possible reason is that our sample’s average weekly duration of outdoor activities was merely 3.695 h, indicating a significant deficiency in outdoor activity time. Insufficient outdoor activity time has been proven to have a significant impact on adolescent health [62–64]. Therefore, due to the EQ-5D-Y-3L describing population health as having more health problems [65], respondents could have substantial fluctuations in their perception of their already low amount of outdoor activity time, resulting in only a moderate correlation between the two scales for the UA dimension.

In terms of known-groups validity, the EQ-5D-3L and EQ-5D-Y-3L exhibit mixed performance. Neither scale demonstrated significant discriminatory capability across demographic characteristics or distinct myopia subgroups. A potential reason for this may be attributed to the homogeneity of the study sample, which was concentrated within the ages of 15–17 years and uniformly comprised of students currently enrolled in school, thereby limiting the scales’ ability to effectively differentiate between various subgroups. However, known-groups validity suggests that both the EQ-5D-3L and EQ-5D-Y-3L are able to differentiate between groups with varying levels of EQ VAS. For the EQ-5D-Y-3L, which has more comprehensible wording, these differences tend to be more pronounced (ES = 1.793 for EQ-5D-3L, ES = 1.920 for EQ-5D-Y-3L). Besides, based on effect sizes, we found that the EQ VAS appears to be more sensitive in utility value. This finding aligns with the previous comparative study on the measurement performance of EQ-5D-Y versus KIDSCREEN‑10 scales among Chinese populations [15]. The better discriminatory power of EQ VAS may be because it makes respondents think about health dimensions beyond those covered by EQ-5D-3L or EQ-5D-Y-3L. A contemporary investigation involving adult participants revealed that numerous health dimensions were taken into account when they responded to the EQ VAS [66]. While a systematic review of summarizing measurement properties of the EQ VAS demonstrated that the EQ VAS exhibits “sufficient” construct validity, “inconsistent” test-retest reliability, and “inconsistent” responsiveness across a broad range of populations [67]. Comparable research focusing on adolescent populations is currently inadequate. Therefore, there is a need for studies to assess the EQ VAS as an additional indicator of HRQoL among adolescent populations.

Several limitations in our study should be noted. First, all respondents included in this study were high school students, which may not be fully representative of this age group, potentially affecting the generalizability of the findings. Second, an online survey was used in this study, which may affect the quality of collected data. And we could not reach the potential respondents who do not use the internet. Third, given the concern of cognitive burden and time consumption for adolescents in high school, there was a limited number of characteristics collected in this study. Fourth, this study was conducted based on cross-sectional data rather than longitudinal data. Therefore, it is not possible to assess and compare test-retest reliability and longitudinal responsiveness. Further research is required to further test their measurement properties using longitudinal data. Fifth, we assessed convergent validity by comparing the correlation between EQ-5D-3L and EQ-5D-Y-3L. Although there is no gold standard for measuring HRQoL in children and adolescents, using an external instrument as a benchmark to evaluate convergent validity may provide more informative results.

## Conclusions

Both the EQ-5D-3L and EQ-5D-Y-3L have shown comparable validity and sensitivity among adolescents aged 15–17 years in China. Both measures also demonstrate generally good convergent validity across the relevant dimensions and their response levels. However, there is a non-negligible proportion of inconsistency in responses of corresponding dimensions between the two measures, especially in PD and AD dimensions. Besides, respondents reported higher proportions of having problems using the EQ-5D-Y-3L than using the EQ-5D-3L. This indicates that the EQ-5D-3L and EQ-5D-Y-3L may not be interchangeable. Further research is needed to compare other measurement properties of these two measures, such as test-retest reliability and longitudinal responsiveness among a representative sample of Chinese adolescents.

## Data Availability

No datasets were generated or analysed during the current study.
